# Supplementary figures and images for: Looking for Visitor’s Effect in Sanctuaries: Implications of Guided Visitor Groups on the Behavior of the Chimpanzees at Fundació Mona
Source: Animals (Basel). 2019 Jun 13;9(6):347. doi: 10.3390/ani9060347 (PMC6617045; doi:10.3390/ani9060347)

Normal Q-Q

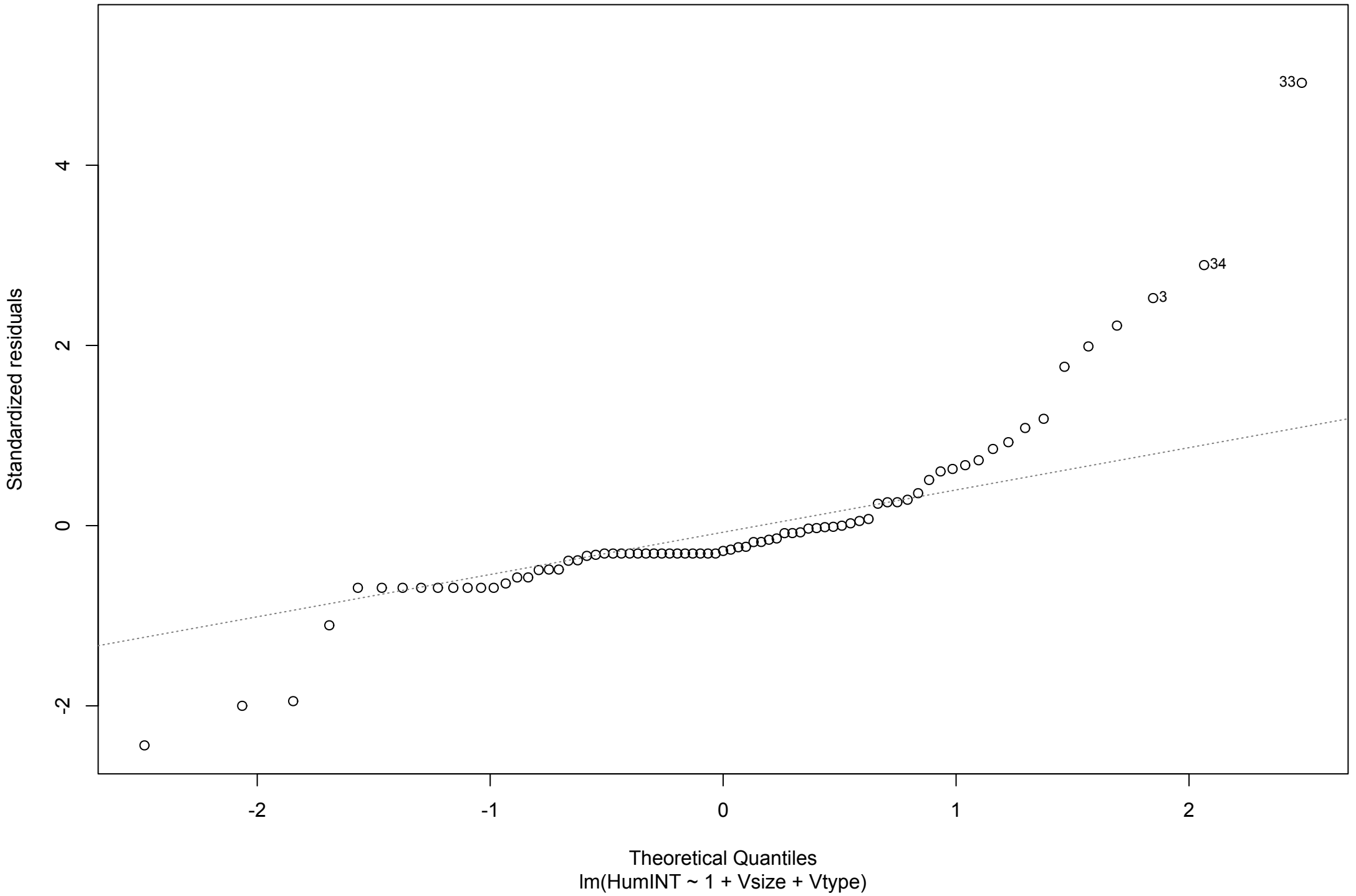

Supplement: Supplementary file 1 [file animals-09-00347-s001.zip › Figure S2. QQ plot residuals model3 human interaction.pdf]

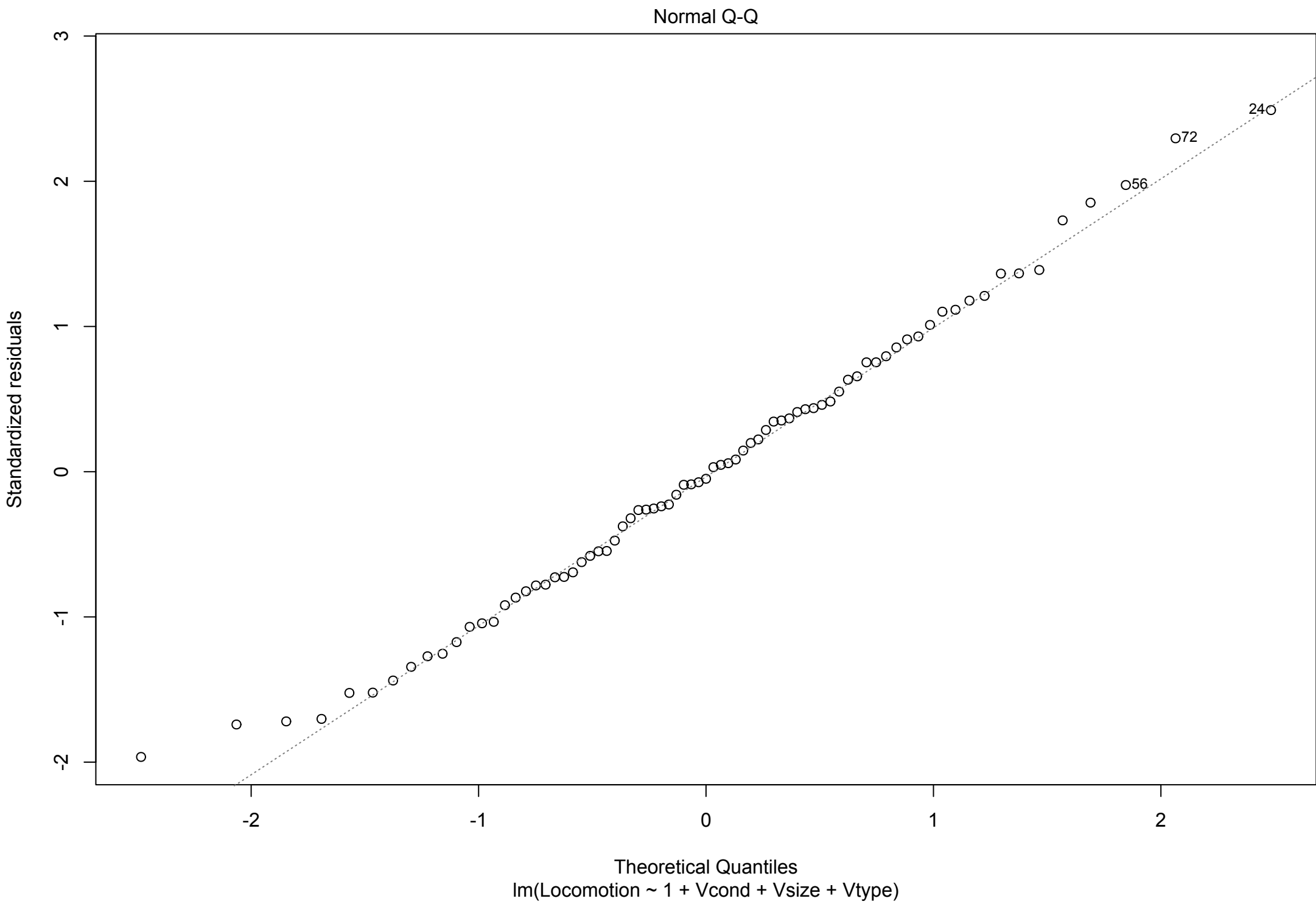

Supplement: Supplementary file 1 [file animals-09-00347-s001.zip › Figure S1. QQ plot residuals model5 locomotion.pdf]
